# Understanding Amyloid Structures and Disease: A Continuing Challenge in Health Research

**DOI:** 10.3390/ijms22126620

**Published:** 2021-06-21

**Authors:** Grazia Chiellini

**Affiliations:** Department of Pathology, University of Pisa, 56100 Pisa, Italy; grazia.chiellini@unipi.it

Neurodegenerative disorders (NDDs), including Alzheimer’s, Parkinson’s, and Huntington’s diseases, are a highly prevalent class of disorders that share the presence of aberrant aggregates called amyloids in the nervous system [1]. Amyloidogenic proteins are relatively small, typically soluble, and intrinsically disordered proteins that undergo remarkable conformational re-arrangements associated with a process of aggregation and self-assembly that ultimately leads to the formation of fibrillar aggregates designated as amyloid fibrils. Even though recent years have witnessed an increasing interest in the self-assembly of amyloidogenic proteins [2], with growing numbers of multidisciplinary scientific approaches, the elucidation of the atomic structure of amyloid fibrils formed from their intact protein precursors and the mechanisms relating fibril formation to disease have remained elusive.

Over the last few decades, there have been extensive efforts to develop novel inhibitors and modulators as potential therapeutics for amyloidogenic protein aggregation and the development of amyloid disease [3]. Up to the present, however, drug therapies have been used to provide almost exclusively symptomatic benefits, and there is still an urgent need for accurate diagnostic approaches and innovative therapeutics able to prevent or delay the onset of the disease. As elegantly reviewed in this Special Issue by Jin et al. [4], amyloidogenic proteins are appealing potential biomarkers for neurodegenerative diseases, but their diagnostic success is intertwined with the development of combined detection strategies, involving other types of biomarkers, organ systems, and ultra-sensitive technologies.

Several studies have shown that transthyretin (TTR), a well-known transporter of thyroid hormones (THs) and vitamin A, is a major binder of amyloid beta (Aβ) in cerebrospinal fluid and plays an essential role in suppressing Aβ aggregation in neurodegenerative pathologies [5]. As well-described by Saponaro et al. [6], the TTR/Aβ complex is emerging as a possible biomarker for Alzheimer’s disease (AD), highlighting the intriguing potential of TTR stabilizers as novel targets for therapeutic intervention in AD.

By combining nuclear magnetic resonance (NMR) spectroscopy, computational simulation, and biochemical assays, Kim et al. [7] found that a small series of diphenyl-methane-based thyroid hormone analogs, including well-characterized lipid lowering agent Sobetirome [8], originally named GC-1, and recently developed TRβ selective agonist IS25 and its prodrug TG68 [9,10] act as TTR stabilizers and efficient suppressors of TTR aggregation, further expanding the potential of thyromimetics as multi-functional therapeutic molecules for TTR-related pathologies, including NDDs.

In addition, Bcl-xL, a pro-survival protein involved in apoptosis regulation [11], has been previously reported to form various types of fibers, from native to amyloid conformations. By developing specific monoclonal antibodies (mAbs) directed against amyloidogenic conformations of Bcl-xL, Gonneaud et al. [12] were able to detect the presence of Bcl-xL in amyloid aggregates in neuroblastoma SH-SY5Y cell lines. Therefore, these mAbs may further help in developing new diagnostics and therapies, by exploiting Bcl-xL as a strategic target for NDDs.

It is important to note that even though the successful application of monoclonal antibodies in AD therapy is still being debated [13], in October 2020, the European Medicines Agency (EMA) accepted Biogen’s monoclonal antibody Aducanumab as a potential AD drug, as did the U.S. Food and Drug Administration (FDA) in June 2021.

As described in Robinson et al.’s report [14], peptide-based Aβ aggregation inhibitors are potential preventative strategies that have some advantages as compared to monoclonal antibodies (mAbs), including low immunological profile, small size, and tunable, drug-like characteristics.

By using mouse hippocampal-derived HT22 cells, the authors showed that two of the tested synthetic pseudo-peptide inhibitors, namely SG inhibitors, developed using a rational drug design approach and predicted to bind Aβ in anti-parallel orientation, demonstrated neuroprotection against Aβ(1–42). On the other hand, a third inhibitor, predicted to bind parallel to Aβ, was not neuroprotective. They also showed that myristoylation of SG inhibitors, intended to enhance delivery across the blood–brain barrier (BBB), resulted in cytotoxicity. Overall, by applying three different methodological approaches, including molecular dynamics simulations, single molecule biophysical studies, and in vitro cell viability assays, Robinson et al. provided fundamental clues to the future development of peptide aggregation inhibitors against Aβ toxicity.

Serum amyloid A (SAA) is one of the most important precursor amyloid proteins and plays a vital step in amyloid A (AA) amyloidosis [15]. In their study, Lin et al. [16] showed that rosmarinic acid (RA), which is a well-known inhibitor of the aggregation of amyloid β (Aβ), displayed inhibitory activity against SAA aggregation in vitro using a microliter-scale high-throughput screening (MSHTS) system with quantum-dot nanoprobes. These findings suggest that the dietary intake of RA enhanced the amyloid aggregation inhibitory activity of blood and suppressed SAA deposition in organs. This study also demonstrated that the MSHTS system could be applied to in vitro screening and to monitor comprehensive activity of metabolized foods adsorbed by blood.

Recent data indicate that molecular chaperones and chaperon-like proteins have the ability to inhibit the formation of pathological amyloid fibrils [17]; therefore, the chaperone-based therapy of amyloidosis has recently been proposed [18]. However, while a large number of studies indicate that these proteins can be specifically and effectively targeted to slow or prevent amyloid disease progression, their effects on mature amyloid fibrils is currently a much less studied problem. Considering that amyloidosis is often detected at late stages of the diseases, when a large number of amyloid plaques have already accumulated in the patient’s body, targeting mature amyloid fibrils is of high importance for the treatment of progressive amyloidosis. In this regard, the work of Stepanenko et al. [19] showed that a heat shock protein, alpha-B-crystallin, which is capable of inhibiting fibrillogenesis and is found in large quantities as a part of amyloid plaques, can induce the degradation of mature amyloids by reducing the ordering of these protein aggregates under physiological conditions. The authors emphasize that the activity of chaperons and chaperon-like proteins has Janus head features, the pathophysiological manifestation of which may depend on the balance of cellular proteostasis. It is therefore necessary to consider that a chaperone-based therapy of amyloidosis might require particular caution.

Even though the involvement of nascent Aβ monomers in the pathological route of AD is currently considered to be the most relevant [20,21], an emerging perspective suggests that nascent Aβ, out of the amyloidogenic pathway, plays a physiological and protective role, especially in the brain [22]. As reported by Rondelli et al. [23], when cleaved from parent amyloid precursor protein (APP), nascent Aβ monomer may interact with nearby membrane environment on its way to the target. Therefore, by using an innovative integrated approach comprising small-angle neutron scattering (SANS), differential scanning calorimetry, X-ray scattering, and neutron reflectometry (NR), the authors were able to observe the details of the interaction of Aβ monomers with membranes, with no invasivity. Notably, their work revealed that the rules of monomer–membrane engagement and the resulting structural effects are dictated by the chemical–physical properties of the membrane rather than by the Aβ peptide variants. Interestingly, they also unveiled an unknown structural role of Aβ monomers in inducing tightening of adjacent complex membranes, thereby affecting a basic structural event for cell–cell adhesion and cell motility.

Recent studies have demonstrated that in response to damaging stimuli, the brain puts in place restoration mechanisms that relay chiefly on the protective function of astrocytes and microglia [24].

As well-described by Lana et al. in their review [25], the concerted actions of astrocytes and microglia in the formation of triads with neurons help recognize danger signals and to dispose of damaged neurons or neuronal debris by phagocytosis. Degenerating neurons are engulfed by microglia, and reactive astrocytes cooperate in the phagocytic event, possibly to prevent the spread of noxious neuronal debris in the tissue. Notably, the mutual interplay between astrocytes and microglia can result in virtuous/vicious cycles which differ in different brain regions. As suggested by Lana et al., a differential reactivity of astrocytes and microglia in CA1 and CA3 areas of the hippocampus in a transgenic mouse model of Aβ deposition (TgCRND8 mice) [26] can be responsible for the differential sensitivity of the two areas to insults.

Therefore, understanding the spatial differences and roles of glia will substantially contribute to assess how these interactions can influence the state and progression of the disease, and will be critical for identifying therapeutic interventions for AD.

In summary, all articles published in this Special Issue provide a significant contribution to the ongoing understanding of amyloid structures and their roles in NDDs.

It is a pleasure for the Guest Editor to gratefully acknowledge all the authors for their important contributions into this Special Issue and the advancement of knowledge for accurate diagnosis and therapeutic interventions.
